# Does an Association Exist Between Food Insecurity and Eating Disorder Symptoms Among Young People Living in England?

**DOI:** 10.1192/bjo.2024.240

**Published:** 2024-08-01

**Authors:** Katie Sampson, Katherine Memory, Mina Fazel, Paul Moran, Helen Bould

**Affiliations:** 1 University of Bristol, Bristol, United Kingdom; 2 University Hospitals Bristol and Weston NHS Foundation Trust, Bristol, United Kingdom; 3 University of Oxford, Oxford, United Kingdom

## Abstract

**Aims:**

Food insecurity, defined as lacking regular access to nutritious food due to financial hardship, is associated with a range of adverse developmental outcomes for children and adolescents. Emerging evidence suggests food insecurity in adults may be associated with disordered eating behaviours, including binge eating and unhealthy weight control strategies. However, the nature of this relationship in adolescents remains unclear. This study aimed to investigate whether an association exists between food insecurity and eating disorder symptomatology in a large and diverse sample of adolescents living in England.

**Methods:**

Cross-sectional data were collected from 34,730 young people in school years 7 to 13 (aged 11 to 18) in classrooms across England, as part of the OxWell 2023 Student Survey. Eating Disorder symptomatology was measured, on a scale of 0 to 6, with five self-report screening questions from the Eating Disorder Section of the Development and Well-Being Assessment (DAWBA) and one additional question on meal skipping due to shape/weight concerns. Food insecurity was measured, on a scale of 0 to 6, with three questions adapted from the Wales Young People's Survey on Child & Family Poverty 2019. A complete case analysis was conducted using Stata, v18. Regression analyses were performed to test for associations between food insecurity and eating disorder symptomatology, stratified by gender and adjusting for age and ethnicity.

**Results:**

12,571 (36.2%) participants were excluded due to missing data in key study variables. Our final sample comprised 22,159 adolescents with a mean age of 13.8 years (50.8% female, 54.4% white ethnicity). 63.6% of participants reported experiencing at least one eating disorder symptom and 45.7% scored ≥2 on the DAWBA screening items, a more stringent cut-off for possible eating disorder. Food insecurity was found to be a significant predictor of eating disorder symptomatology in participants of all genders (female: β 0.54, 95% CI 0.48–0.60, p < 0.001, male: β 0.40, 95% CI 0.36–0.44, p < 0.001, other: β 0.52, 95% CI 0.43–0.61, p < 0.001). The association was particularly marked amongst those reporting purging behaviours (OR 1.62, 95% CI 1.55–1.69, p < 0.001).

**Conclusion:**

In keeping with previous research, our findings indicate that adolescents experiencing food insecurity exhibit increased rates of eating disorder symptomatology. Further research is needed to explore potential mechanisms behind this association, as well as to develop effective intervention strategies. Our study adds to a body of evidence identifying a high-risk and disenfranchised group of young people who may benefit from targeted support.

